# Effect of erythritol and xylitol on *Streptococcus pyogenes* causing peritonsillar abscesses

**DOI:** 10.1038/s41598-021-95367-y

**Published:** 2021-08-04

**Authors:** Siiri Kõljalg, Risto Vaikjärv, Imbi Smidt, Tiiu Rööp, Anirikh Chakrabarti, Priit Kasenõmm, Reet Mändar

**Affiliations:** 1 Department of Microbiology, Institute of Biomedicine and Translational Medicine, University of Tartu, Tartu, Estonia; 2 Department of Oto-Rhino-Laryngology, Institute of Clinical Medicine, University of Tartu, Tartu, Estonia; 3 Ear-Nose-Throat Clinic, Tallinn, Estonia; 4 Competence Centre on Health Technologies, Tartu, Estonia; 5 Cargill R&D Centre Europe BVBA, Havenstraat 84, 1800 Vilvoorde, Belgium

**Keywords:** Microbiology, Diseases, Health care, Medical research

## Abstract

Polyols are effective against caries-causing streptococci but the effect on oropharynx-derived pyogenic streptococci is not well characterised. We aimed to study the effect of erythritol (ERY) and xylitol (XYL) against *Streptococcus pyogenes* isolated from peritonsillar abscesses (PTA). We used 31 clinical isolates and 5 throat culture collection strains. Inhibition of bacterial growth by polyols at 2.5%, 5% and 10% concentrations was studied and the results were scored. Amylase levels in PTA pus were compared to polyol effectivity scores (PES). Growth curves of four *S. pyogenes* isolates were analysed. Our study showed that XYL was more effective than ERY inhibiting 71–97% and 48–84% of isolates, respectively, depending of concentrations. 48% of clinical and all throat strains were inhibited by polyols in all concentrations (PES 3). PES was negative or zero in 26% of the isolates in the presence of ERY and in 19% of XYL. ERY enhanced the growth of *S. pyogenes* isolated from pus with high amylase levels. Polyols in all concentrations inhibited the growth in exponential phase. In conclusion, ERY and XYL are potent growth inhibitors of *S. pyogenes* isolated from PTA. Therefore, ERY and XYL may have potential in preventing PTA in the patients with frequent tonsillitis episodes.

## Introduction

Peritonsillar abscess (PTA) is a collection of pus between the fibrous capsule of the palatine tonsil and the superior pharyngeal constrictor muscle^1^. PTA can be a complication of acute tonsillitis^2^. In case of minor salivary gland involvement in PTA pathogenesis, amylase, the enzyme present in human saliva, is detected in high levels in peritonsillar pus^3^. PTA is one of the most common diseases in ear, nose and throat department requiring an emergency hospitalization. This disease is prevalent in adolescents and young adults^4, 5^.

PTA is usually diagnosed based on clinical signs such as unilateral peritonsillar swelling, medially displaced tonsil and deviation of uvula toward unaffected side^6^. Airway obstruction and abscess extension into deeper neck spaces are severe but infrequent complications of PTA^7^. However, patients with PTA, especially children and older adults, are found to be significantly ill, uncomfortable, in pain and dehydrated.

The common bacterial pathogen causing PTA is *Streptococcus pyogenes*^8^. *S. pyogenes* is the most common species in the genus *Streptococcus* being first described in 1884 by Rosenbach. They are *β*-haemolytic Gram positive cocci arranged in chains and belong to Lancefield group A. *S. pyogenes* strains are genetically diverse and can cause large variety of pyogenic and non-pyogenic infections with a mild to extremely severe course of the disease^9^.

Polyols, like xylitol (XYL) and erythritol (ERY) are sugar alcohols not metabolized in the body, and used widely in food industry. Clinical investigations have shown that both xylitol, a pentitol type sugar alcohol, and erythritol, a tetritol-type alditol to be effective against cariogenic and periodontogenic bacteria i.e. *S. mutans, S. sobrinus, S. gordonii, Porphyromonas gingivalis, Scardovia wiggsiae*^10–13^. While xylitol has been shown to have inhibitory effect against *lactobacilli*^14^, to impact biofilm formation of *Staphylococcus aureus* and *Pseudomonas aeruginosa*^15^, and erythritol to play role in *Brucella melitensis* virulence^16^, characterization of the potential effect of polyols against important oropharynx derived pathogens such as *S. pyogenes*, is insufficient so far.

We therefore aimed to evaluate the effect of erythritol and xylitol against *S. pyogenes* isolated from peritonsillar abscesses.

## Results

### Growth characteristics of *S. pyogenes* PTA isolates in the presence of polyols after 24-h incubation

**Figure 1:**
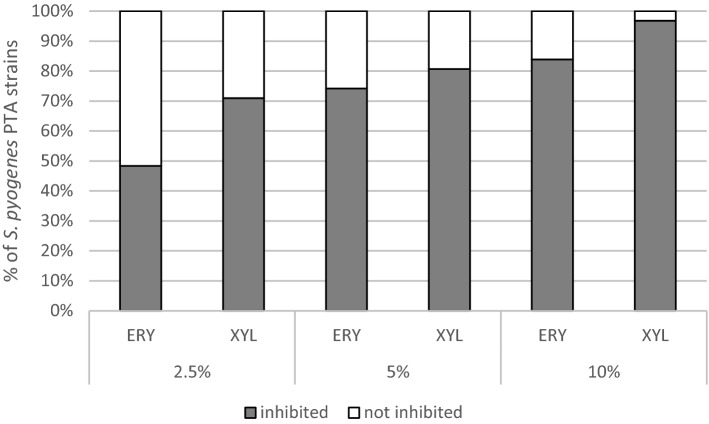
Effect of different concentrations (2.5%, 5% and 10%) of erythritol ERY and xylitol XYL on the growth of *S. pyogenes* PTA isolates (n = 31).

**Figure 2:**
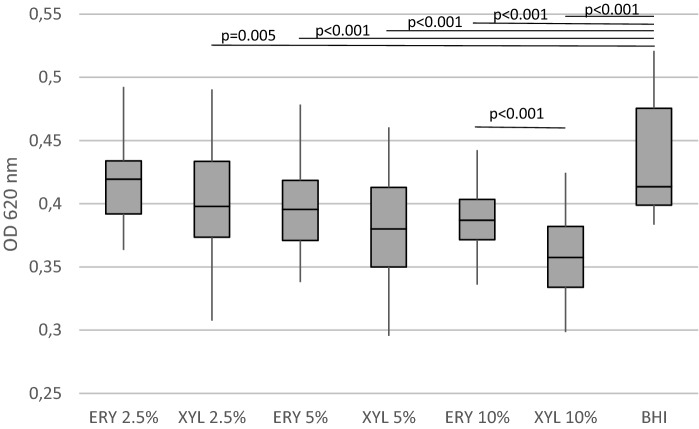
The growth of 31 *S. pyogenes* isolates from PTA pus in the presence of ERY erythritol, XYL xylitol and BHI brain heart infusion. Minimal, maximal, median, 25 percentile and 75 percentile values of optical density are presented.

The impact of ERY and XYL in different concentrations (2.5%, 5% and 10%) was studied on 31 *S. pyogenes* isolates from PTA pus and five type collection strains with throat origin and the results were compared with the growth in BHI. Overall, xylitol was more effective and inhibited the growth in 71–97% of investigated PTA isolates, while erythritol was less active inhibiting 48–84% of investigated isolates depending of concentrations (Fig. 1). 10% of polyol solution was the most active, followed by 5% and 2.5%. (Fig. 2). All tested polyol concentrations, except ERY 2.5%, showed statistically relevant (p < 0.0001–0.009) inhibitory effect against PTA isolates compared to BHI. 10% xylitol was more effective than 10% erythritol (p = 0.0005). The growth of all throat derived type collection strains was inhibited by both polyols in all studied concentrations.

**Figure 3:**
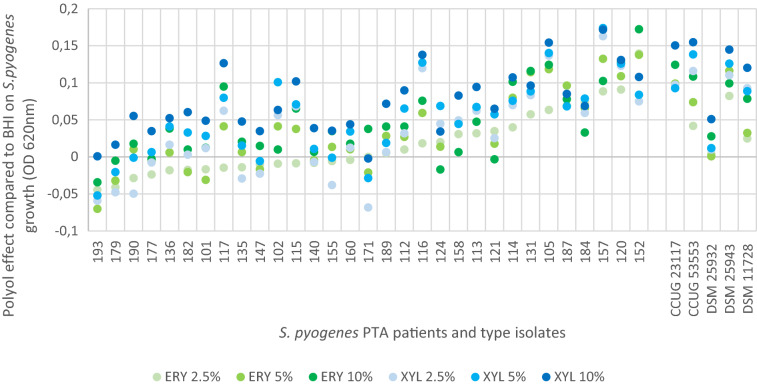
Effect of erythritol and xylitol on *S. pyogenes* PTA isolates and type strains compared to BHI. For each condition (e.g. for polyol ERY at 2.5%), the difference between the polyol growth data and BHI growth data (OD value of BHI minus OD value of polyol solution) is represented. The colored circles above the value “0” in Y axis are “inhibited growth”, values below the value “0” are enhanced growth, and on the value “0” is “no change”. The growth inhibition was detected in 28/31 of the isolates in the presence of at least one concentration of ERY and in 30/31 isolates of XYL.

As there were inter-isolate differences in the impact of the polyols, polyol effect was analysed for each *S. pyogenes* isolate. The impact of different concentrations of ERY and XYL, on the growth of studied *S. pyogenes* isolates compared to BHI, was calculated as the difference between the polyol growth data and BHI growth data for each isolate. Respective results are presented in Fig. 3. Both polyols showed a high inhibitory effect against most of the isolates. The growth inhibition was detected in nine tenth (28/31; 90%) of the isolates in the presence of at least one concentration of ERY and in all except one (97%) isolate of XYL. However, in 18/31 (58%) of isolates ERY and in 9/31 (29%) of isolates XYL led to enhanced growth of *S. pyogenes* PTA isolates in at least one studied concentration after 24 h incubation.

### Polyol inhibition effectivity scores

**Figure 4:**
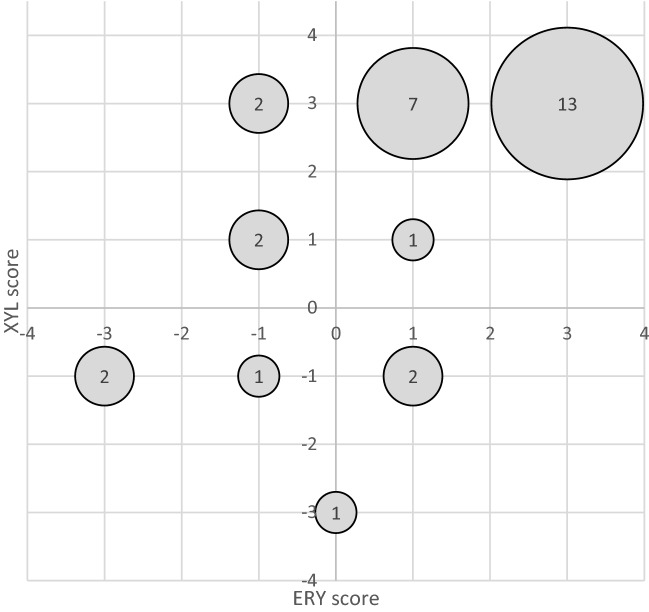
Distribution of *S. pyogenes* PTA isolates based on combined polyol effectivity scores (PES) for ERY and XYL. PES was calculated as a summary effect of three polyol concentrations (2.5%, 5% and 10%), where each polyol concentration contributed − 1, 0 or + 1 points depending on whether the *S. pyogenes* isolate growth increased, remained unchanged or decreased in the presence of the polyols, respectively. Size of the bubble represents number of isolates with different scores.

Polyol effectivity scores (PES) were calculated to summarize the inhibitory effect of polyols in different concentrations (2.5%, 5% and 10%) for each isolate. PES was calculated as a summary effect of three polyol concentrations (2.5%, 5% and 10%), where each polyol concentration contributed − 1, 0 or + 1 points to the PES depending on whether the *S. pyogenes* isolate growth increased, remained unchanged or decreased in the presence of the polyols, respectively (Fig. 4). For example, if for an isolate X, all the three concentrations of XYL led to decrease in growth, XYL would have a PES score of 3 (+ 1 points for each concentration leading to decreased growth), while if two concentrations of XYL led to decrease and one led to increase in growth, XYL would have a PES score of 1 (which is a cumulation of + 2 points for two concentrations leading to decreased growth and -1 point for one leading to increased growth). Almost half (13/31; 42%) of *S. pyogenes* PTA isolates and all throat strains from the culture collection were inhibited by ERY and XYL in all the studied concentrations (as indicated by polyol effectivity scores 3). The polyol effectivity score was negative or zero for around a quarter (8/31; 26%) of the isolates in the presence of ERY and for one fifth (6/31; 19%) of the isolates in the presence of XYL. For 24 isolates, XYL and ERY impact as assessed by the PES was similar (Fig. 4), i.e. positive PES for both XYL and ERY or negative for both XYL and ERY. For 6 isolates, PES scores indicate potentially opposing impacts of ERY and XYL (Fig. 4).

### Polyol effectivity score against *S. pyogenes* isolates from PTA pus depending on amylase levels in pus

**Figure 5:**
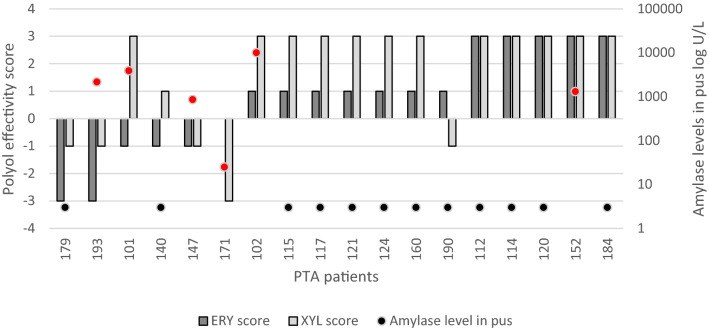
Amylase levels in PTA pus (red dots—high level, black dots—low level) and polyol effectivity score (PES) against *S. pyogenes* isolates from PTA pus. PES was calculated as a summary effect of three polyol concentrations (2.5%, 5% and 10%), where each polyol concentration contributed − 1, 0 or + 1 points depending on whether the *S. pyogenes* growth increased, remained unchanged or decreased in the presence of the polyols, respectively.

High amylase level in PTA pus indicates association with Weber’s salivary gland infection. Among the patients from where the *S. pyogenes* was isolated, corresponding data about the respective amylase levels in PTA pus was available for 18 patients. High amylase levels (25–10,019, median 860 U/L) were detected in 6 patients and low amylase levels (< 3 U/L) were detected in 12 patients. ERY inhibitory score of isolated *S. pyogenes* isolates was negative or zero in two third of high pus amylase cases compared to 17% of low pus amylase cases (4/6 vs 2/12, respectively; p = 0.034). XYL inhibitory score was negative in half of high pus amylase cases (3/6) compared to 17% of low pus amylase cases (3/6 vs 2/12, respectively; p = 0.139) (Fig. 5).

### Growth curves of *S. pyogenes*

**Figure 6:**
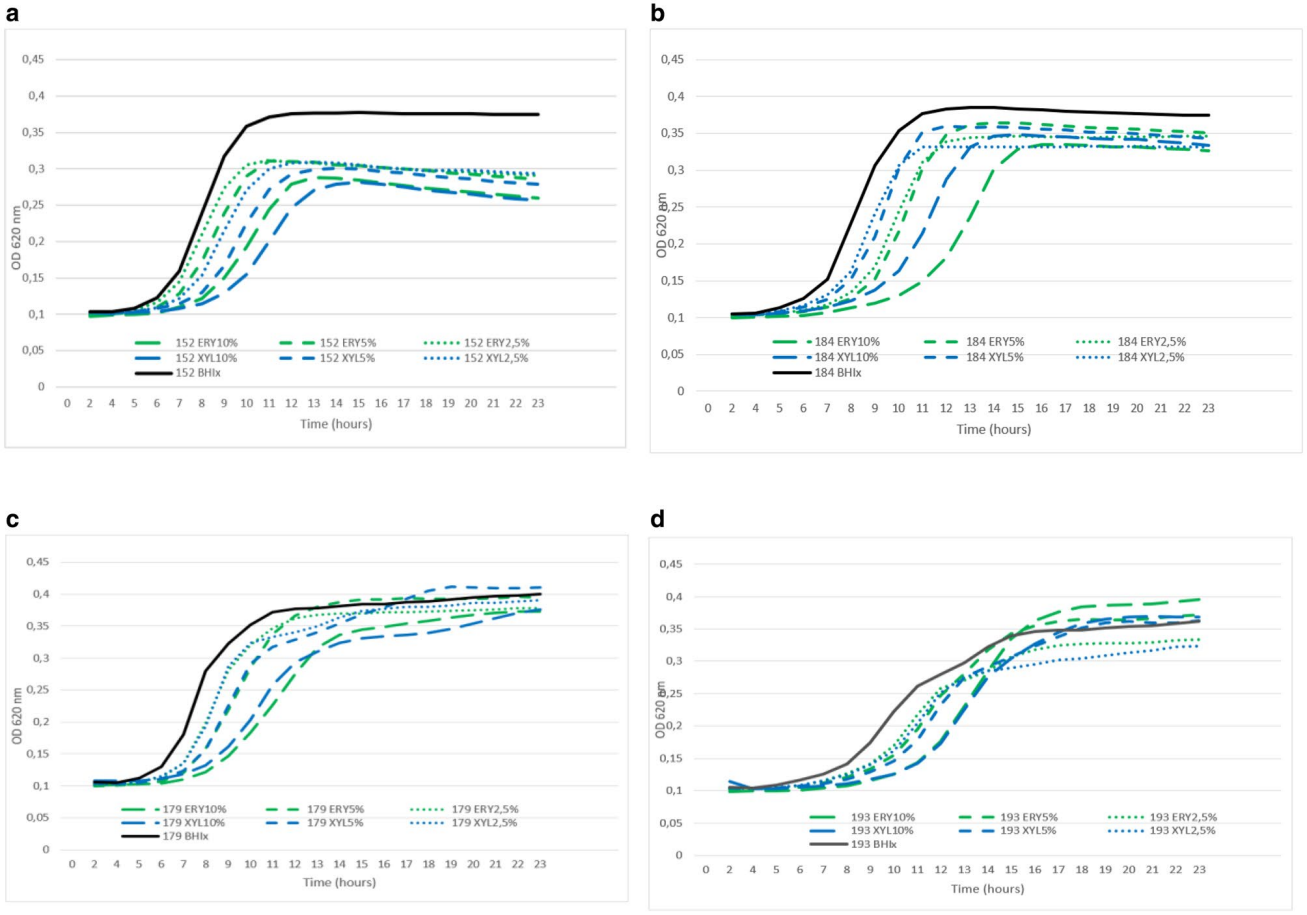
*S. pyogenes* growth curves with ERY erythritol, XYL xylitol and BHI brain heart infusion. Isolates with high polyol effectivity scores (**a**) *S. pyogenes* 152, (**b**) *S. pyogenes* 184 and low polyol effectivity scores (**c**) *S. pyogenes* 179, (**d**) *S. pyogenes* 193.

The growth of four *S. pyogenes* isolates *S. pyogenes* 152, *S. pyogenes* 184, *S. pyogenes* 179 and *S. pyogenes* 193 (selection of isolates detailed in materials and methods) was monitored from 0 to 24 h. Significant growth was detected after 7 h of incubation and *S. pyogenes* isolates reached to stationary phase after 10 to 13 h of incubation. Strains with high polyol effectivity scores (*S. pyogenes* 152 and *S. pyogenes* 184; Fig. 6a,b) and low polyol effectivity scores (*S. pyogenes* 179 and *S. pyogenes* 193; Fig. 6c,d) in 24-h study showed similarly good effectivity of erythritol and xylitol in exponential growth phase compared to BHI in growth curve study. Both the polyols, consistently inhibited the growth of all studied isolates in all studied concentrations in exponential growth phase.

## Discussion

Our study showed that ERY and XYL influences the growth of *S. pyogenes* isolated from PTA in a strain-specific manner. In the exponential growth phase, both the polyols, in all the tested concentrations, were effective against *S. pyogenes*, however in stationary phase the effect of polyols was type and concentration dependent. The growth inhibition was detected in nine tenth of the isolates in the presence of at least one concentration of ERY and in all except one isolate of XYL. *S. pyogenes* from the patients with high amylase levels in the PTA pus, indicating small salivary glands as possible source of PTA, were less responsive to growth inhibitory effect of ERY as opposed to XYL.

ERY and XYL have been proven to inhibit the growth of oral cariogenic bacteria and widely used in caries prophylactics^11, 17^. Strain-specific effect of polyols on cariogenic bacteria such as *S. mutans, S. sobrinus* and *S. wiggsiae* has been demonstrated in previous studies^10, 18^. As there are inter-strain differences among bacterial species, research conclusions based on observations from a large number of isolates should be more reliable and translatable. The results presented in this study is a cumulation of experiments on 36 *S. pyogenes* isolates, including 31 clinical *S. pyogenes* isolates and five throat derived strains from the culture collections. To our best knowledge, this is the first study to address polyol efficacy on the oropharynx derived pathogen, *S. pyogenes*, which is able to cause acute tonsillitis and potentially life-threatening PTA. ERY has been shown to be more effective than XYL against caries causing oral mutans streptococci^11, 12, 17^. Though both polyols are generally similar, they differ in size and in the effectivity to scavenge hydroxyl radicals. Accordingly, superiority of ERY against cariogenic streptococci is associated with smaller molecule size and higher biological activity^19^. In our present study, we found XYL to be more effective against *S. pyogenes* than ERY, even though *S. pyogenes* and mutans streptococci are genetically closely related. Further research is needed to clarify the exact mechanisms behind these findings.

Erythritol and xylitol are generally considered non-toxic and safe for human consumption^11^. At the same time, they inhibit the growth of bacteria. Antibacterial effect of xylitol has been associated with accumulation of toxic nonmetabolizable xylitol phosphate in the bacterial cells inhibiting glucose uptake and glycolytic enzymes of bacteria^20, 21^. Inactivation of the *fxpC* gene coding for the fructose- phosphoenolpyruvate:sugar phosphotransferase system gives resistance to xylitol in *S. mutans*^21^. Erythritol mechanism of action is considered similar to xylitol^22, 23^, however exact reasons for non-susceptibility are not clarified yet.

Previous investigations have shown that polyols, and also triclosan, may in certain concentrations enhance the biofilm formation of bacteria instead of inhibition^10, 24^. In our study we observed both, growth inhibition and enhancement depending on polyol concentration and specificity of the *S. pyogenes* isolate. In order to compare the isolates and to generalize the results, we scored and summed three polyol concentration results according to the effect to the growth (positive effect, zero effect or negative effect). The growth of all throat-derived type collection strains and half of the PTA isolates was inhibited in the presence of all polyol concentrations used in our study (2.5%, 5% and 10%) in a 24 h challenge. The growth of another half of clinical PTA isolates was enhanced by all or some polyol concentrations. The matter of differences in polyol concentrations in various oropharynx locations must be considered. Though the safety of polyol consumption in the acute phase of infection with high rate of *S. pyogenes* replication, the possibility that under certain conditions the polyols may promote growth of potential pyogenic pathogen, should not be overlooked.

We were interested in linking the differences of the clinical attributes of the patients and the corresponding behaviours of *S. pyogenes* isolated from the patients. Amylase is an enzyme found in human saliva and increased amylase levels in PTA pus are associated with involvement of minor salivary glands in infectious processes^3, 8^. High amylase in PTA abscess indicates inflammation of the salivary gland as the onset of infection. We tried to reveal possible differences between the isolates originating from the patients with high and low amylase levels. The amylase levels in pus were available from 18 patients. Erythritol had limited growth inhibitory effects (growth either increased or remained unchanged) on *S. pyogenes* isolated from pus with high amylase levels. The similar effect of xylitol was not statistically significant.

In the rapid growth (exponential growth) phase of *S. pyogenes*, polyols were effective in all concentrations against all studied isolates. Loimaranta et al.^18^ have also found the inhibitory effect of polyols on *S. mutans* biofilm formation to be more noticeable in the early stages of growth. The highest concentrations (10% ERY and 10% XYL) were the most effective in the rapid growth phase. We found that the growth of *S. pyogenes* may be promoted by polyols at certain concentrations. For peritonitis caused by *E. coli*, animal experiments have shown that in the stationary phase of bacterial growth, slow bacterial growth actually occurs in parallel with the non-multiplying bacteria and the destruction of bacteria by the body's defences ^26^. Laboratory experiments have shown that *S. pyogenes* strains can remain in saliva for a long time in the stationary phase ^27^. When using polyols, phase, strain and concentration specific effects would be relevant to take into account.

One limitation of our study is the absence of amylase test results about all PTA cases. However, comparative data of 18 patient’s pus amylase levels and growth characteristics of *S. pyogenes* isolates from the respective pus in the presence of polyols indicate possible differences between PTA cases depending on pathogenesis.

## Conclusion

In conclusion, ERY and XYL are potent growth inhibitors of *S. pyogenes* isolated from PTA patients, especially in the exponential growth phase, though the activity is strain-specific. Therefore, the ERY and XYL-containing candies and chewing gums may have potential in preventing PTA in the patients with frequent tonsillitis episodes.

## Materials and methods

### Chemicals and isolates

Erythritol (Cargill R&D Centre Europe, Vilvoorde, Belgium) and xylitol (≥ 99%, Sigma-Aldrich Co, St. Louis, USA) were tested.

Altogether 36 *Streptococcus pyogenes* isolates, 31 from pus of PTA patients and 5 throat strains from culture collections (CCUG 23117, CCUG 53553, DSM 25932, DSM 25943 and DSM 11728) were included in the study.

The clinical *S. pyogenes* isolates and pus amylase data of respective patients were acquired during prospective clinical study of etiopathogenesis of PTA. The informed consent was obtained from all study subject or, if subjects are under 18, from a parent and/or legal guardian. All samples and data from patients were collected in accordance with relevant guidelines and regulations and all study protocols were approved by an institutional committee (approved by Ethics Review Committee on Human Research of the University of Tartu, protocol No. 255/T-1). All. *S. pyogenes* isolates are deposited in the Human Microbiota Biobank of Tartu (HUMB; http://www.eemb.ut.ee/eng/). Details of the study are described elsewhere^25^.

### Bacterial growth inhibition after 24 h of incubation by different polyol concentrations

The effect of polyols was studied using a previously described method^22, 23^ with slight modifications. Briefly, the tested substances were sterilized by filtration at desired concentration and added to the brain–heart infusion (BHI, Oxoid Limited, Basingstoke, Hampshire, UK) medium (sterilization by autoclaving at 121 °C for 15 min). The bacteria were incubated at 37 °C in 10% CO_2_ for 24 h on blood agar (Oxoid Limited, Basingstoke, Hampshire, UK).

The microtiter plate wells (CELLSTAR 96 well polystyrene suspension culture microplates, F-bottom, Greiner Bio-One GmbH, Kremsmünster, Austria) were inoculated with equal amounts (200 µl) of bacterial (the final test-concentration of 10^5^ CFU/ml) and polyol solution. The bacterial cells were grown at 37 °C in 10% CO_2_ on the microtiter plate. The density of bacterial growth was detected spectrophotometrically in an absorbance microplate reader (Labsystems Multiskan Mcc/340, Fisher Scientific, Pittsburgh, PA, USA) at 620 nm. The measurement time points included 0 and 24 h.

The tested polyol concentrations (weight/volume) included 2.5%, 5% and 10%. The polyol effect was calculated as difference between average BHI and polyol test values. Polyol effectivity scores were calculated as a summary effect of three polyol concentrations (2.5%, 5% and 10%) where each polyol concentration gave − 1, 0 or + 1 points depending on whether the *S. pyogenes* growth increased, remained unchanged or decreased in the presence of polyols compared to the growth in BHI, respectively.

### The growth curve study

Four *S. pyogenes* isolates were selected for the growth curve study, two (*S. pyogenes* 152, *S. pyogenes* 184) with high polyol effectivity scores (ERY score 3, XYL score 3) and two (*S. pyogenes* 179, *S. pyogenes* 193) with low polyol effectivity scores (ERY score − 3, XYL score − 1). One of the high score and one of the low score isolates (*S. pyogenes* 152, *S. pyogenes* 193) were from high amylase pus and one of the high score and one of the low score isolates (*S. pyogenes* 184, *S. pyogenes* 179) were from low amylase pus. For growth curve study, the microtiter plates were prepared similar to growth inhibition study. Following incubation at 37 °C took place in spectrophotometer with integrated incubator (Multiskan Go 1510-00361, Thermo Fisher Scientific Oy, Vantaa, Finland. Skanit Software 3.2) in aerobic conditions. The density of bacterial growth in microwells was detected spectrophotometrically at 620 nm in 1-h interval from 0 to 24 h with preceding shaking of 5 s.

### Statistical analysis

All experiments were performed in triplicate and repeated twice. Each value represents the average of two median results from triplicate experiments. Statistical significance was determined using Chi-square-test and Mann–Whitney test (program PAST version 2.17c). *P*-values < 0.05 were accepted as significant. Figures were made using Microsoft Office Excel software ver. 2016.

## Data Availability

The datasets generated during and/or analysed during the current study are available from the corresponding author on reasonable request.
